# Experimental, predictive and RSM studies of H_2_ production using Ag-La-CaTiO_3_ for water-splitting under visible light

**DOI:** 10.1038/s41598-024-51219-z

**Published:** 2024-01-10

**Authors:** Safaa Ragab, Marwa R. Elkatory, Mohamed A. Hassaan, Ahmed El Nemr

**Affiliations:** 1 National Institute of Oceanography and Fisheries (NIOF), Kayet Bey, El-Anfoushy, Alexandria, Egypt; 2 Advanced Technology and New Materials Research Institute, SRTA-City, New Borg El-Arab City 21934, Alexandria, Egypt

**Keywords:** Hydrogen energy, Catalysis

## Abstract

Ag-La-CaTiO_3_ was used in place of sacrificial agents to assess the influence of operational factors on hydrogen generation in a photocatalytic water splitting system. After being synthesized, the physicochemical features of this substance were accurately described. Several characterization techniques including UV–Vis spectroscopy, FTIR, XRD, XPS, EDX, SEM, TGA, DRS and BET were applied to study the prepared Ag-La-CaTiO_3_ photocatalyst. Ag-La-CaTiO_3_ shows a band in the visible wavelength between 400 and 800 nm at < 560 nm compared to the main CaTiO_3_ band at 350 nm. Ag 4d5s electrons transition to the conduction band (CB), which is responsible for the absorption band at ~ 560 nm (> 2.21 eV). The effects of catalyst concentration, light intensity, and beginning solution pH on the H_2_ generation rate may all be evaluated simultaneously using experimental design procedures. Up to a maximum threshold, where a drop in the rate of gas evolution occurs, it was confirmed that the increase in catalyst dose positively affects system productivity. The initial solution pH plays a crucial role in H_2_ production, and pH = 4 and 10 are the optimum pH with a higher yield of H_2_ production. The highest total H_2_ production rate, 6246.09 μmol, was obtained using a catalyst concentration of 700 mg and solution pH equal to 10 under 1200 W Vis lamp for 3 h. For prediction and optimization, a D-Optimal design was applied and the optimal results were pH 4, the catalyst dose of 645.578 mg and 1200 W with H_2_ production of 6031.11 μmol.

## Introduction

Although hydrogen is used extensively in many industrial processes and is one of the most plentiful chemicals in the universe, it may also be a priceless renewable energy source^1^. Hydrogen has several well-known benefits, two of which are its high energy content per mass and its ease of conversion into other forms of energy^2^. Because the combustion process of direct hydrogen produces only one waste, water, this form of fuel is very intriguing. One of the most exciting methods for producing this alternative energy source is the photocatalytic synthesis of hydrogen from water, which also presents an intriguing means of storing solar energy without requiring its conversion to electricity^3,4^. In the existence of catalysts and a light source, the H_2_O molecule splits during this reaction, liberating H_2_ and O_2_^5^. Despite the obvious benefits of using photocatalytic water splitting to produce H_2_, this method is currently regarded as insufficiently efficient for large-scale use^6,7^. One key element in enhancing this process is said to be the formation of the catalyst with increased activity. Many strategies may be utilized to enhance the photocatalytic activity of semiconductors in water splitting, including the application of microporous supports and metallic catalysts^8,9^. Water-splitting by photocatalysis is a heterogeneous catalytic process, and one of its key components is adsorption. The total rate of the water-splitting process for the creation of hydrogen is largely dependent on the adsorption of water on the photocatalysts' active sites and the length of time the water is retained there^10–12^.

Increasing a process's performance to extract the most possible value from a system, procedure, or end result is known as optimization^13,14^. It is evident from several researches in the literature that a wide range of parameters may effectively produce hydrogen^15–20^. Response surface methodology (RSM), genetic algorithms (GAs), artificial neural networks (ANNs), ANYSYS, and other techniques are utilized in the optimization process^21^. However, RSM is a technique that is frequently chosen because it simultaneously evaluates the interaction and individual effects of the chosen independent parameters. Additionally, because RSM uses less chemicals, time, and tests, it is a realistic technique^22^. A useful tool for various sectors' optimization is the RSM model. Complex process development, enhancement, and optimization are accomplished with RSM^23–26^. The output responses are shown graphically as contour plots, which aid in visualizing the response surface's shape, or as a three-dimensional response surface plot. The impact of different experimental settings on the determined responses may be established with a relatively short number of tests utilizing D-Optimal and Box-Behnken experimental designs, which use fewer design points. Process optimization may be accomplished more rationally and conveniently through experimentation when using the Box-Behnken experimental design^27^.

Numerous publications indicate that semiconductor electrodes and doped photocatalysts, such as TiO_2_^28–30^ and SrTiO_3_^31–33^, react to visible light. One of the most well-known wide-gap oxides is CaTiO_3_, also known by its mineral name, perovskite. CaTiO_3_ has an Eg of around 3.5 eV and is an insulator. But it becomes conductive with appropriate donor-doping^34^. We considered using the material as a photocatalyst in a wet environment because of its low cost, simplicity of production, and exceptionally good chemical stability against acids^35^. The effects of lanthanum and sliver codoping on the photocatalytic capabilities of perovskite CaTiO_3_ are the main topic of this article. When CaTiO_3_ is doped with Ag and La ions in modest amounts, it becomes responsive to visible light. However, CaTiO_3_ is only active when exposed to UV radiation. In this work, visible light was used to examine the band structures and photocatalytic characteristics of the Ag-La codoped CaTiO_3_ and CaTiO_3_ perovskite-type materials. By examining the optical and structural characteristics, the prepared photocatalysts were characterized. After that, the photocatalytic activities were examined in the presence of an obvious light source, and the amount of hydrogen that was created was calculated. Finally, the D-Optimal model was applied in this work to design, predict and optimize the hydrogen production by studying 3 factors (Light intensity, photocatalyst load and pH).

## Materials and methods

### Materials and catalyst preparation of Ag-La-CaTiO_3_ catalyst

Titanium tetraisopropoxide (TTIP), cobalt acetate tetrahydrate, La(NO_3_)_2,_ Ag(NO_3_)_2_, Ca(NO_3_)_2_, citric acid and ethanol were obtained from Sigma Aldrich, USA. Ca_0.94_Ag_0.03_La_0.03_ was prepared from TTIP, Ca(NO_3_)_2_, La(NO_3_)_2_ and Ag(NO_3_)_2_ using the sol–gel method^10^, 3.033 mL of TTIP was added to 20 mL of absolute ethanol (99.9%) with vigorous stirring at room temperature for 30 min, then 5 mL of citric acid as a chelating agent was added to TTIP solution with continuous strong stirring at room temperature for another 30 min. Then, the mixed stoichiometrically solution (3% mole) of Ca(NO_3_)_2_, La(NO_3_)_2_ and Ag(NO_3_)_2_ was added drop-wise slowly to the above TTIP solution with keeping stirring at 50 °C until the solutions became viscous, then the formed yellow solution was moved to an oven and left for drying for 12 h at 50 °C to obtain the xerogel. After the yellow xerogel was produced, excess organic chemicals and nitric acid were eliminated by burning it using a self-spread method. The burning remains were calcined at 850 °C for 10 h to obtain Ag-La-CaTiO_3_. The CaTiO_3_ was prepared in the same manner without adding La(NO_3_)_2_ and Ag(NO_3_)_2_ solutions^10^_._ Schematic of synthesis procedures of Ag-La-CaTiO_3_ photocatalyst are shown in Fig. S1.

### Characterization techniques

The following instruments were applied to identify the samples of Ag-La-CaTiO_3_ and CaTiO_3_ photocatalysts. Ag-La-CaTiO_3_ and CaTiO_3_ NPs crystallinity and average crystal size were confirmed by Meas Srv XRD (D2-diffractometer, Bruker, Germany, that controls at 30 kV, 10 mA using Cu tube of *λ* = 1.5418 Å and 2*θ* with a temperature range of 5° to 80°) was used. Fourier transform infrared (FTIR) spectroscopy model VERTEX70 linked to Platinum ATR model V-100, Bruker, Germany, in the 400–4000 cm^−1^ wavenumber range. SEM (SEM-JEOL, IT 200 Japan) equipped with Energy dispersive X-ray spectroscopy (EDX) was applied to conclude the elemental analysis, materials' morphology and surface characteristics. UV–Visible, GBC Cintra 3030 at the range 190–900 nm spectrophotometer was used to measure the optical absorbance of these samples. The BELSORP-Mini II from BEL Japan, Inc., was applied to measure the mean pore diameter and specific surface area (BET, Brunauer Emmett-Teller). The SDT650-Simultaneous Thermal analyzer equipment was used to conduct thermal analyses of samples utilizing a 10 °C per minute ramping temperature. XPS analysis was conducted on K-ALPHA (Thermo Fisher Scientific, USA) with monochromatic X-ray Al K-alpha radiation − 10 to 1350 eV spot size 400 µm at pressure 10^−9^ mbar with full spectrum pass energy 200 eV and at a narrow spectrum of 50 eV.

### Photocatalytic activity test

The lab-made closed gas system used for the photocatalytic H_2_ generation trials was kept at room temperature. To achieve visible light irradiation, the photoreaction system was typically outfitted with a 1200 W metal halide lamp. The system operated by using a specified quantity of Ag-La-CaTiO_3_ as a photocatalyst in 1000 mL of water without the need for any sacrificial agent. To determine the best catalyst performance and reach the optimum conditions, different Ag-La-CaTiO_3_ photocatalyst dosages (500–800 mg), different working pH at different light sources power (400, 800 and 1200 W) was employed. A magnetic stirrer was applied to continually mix the aqueous solution containing the photocatalyst during the whole reaction phase. The developed gas was initially collected by moving water downhill after passing through an oxygen trap that was filled with an alkaline pyrogallate solution. After that, the amount of gas produced was measured over time and compiled into a data sheet^11^. The generated hydrogen gas was measured by the water displacement method. The optimization of parameters for photodegradation namely, pH, catalyst dosages, and light intensity were performed to find out the best conditions for the efficient photocatalytic degradation.

### Optimization study response surface methodology (RSM)

Response surface methodology (RSM) is a combination of statistical and mathematical techniques used in modelling, prediction, and optimization^10,36,37^. It is feasible to create mathematical models based on the experimental data that is already available thanks to the historical data design and ideal custom design of RSM^10^. The experiment design process used a three-level, three-factor D-optimal. The parameters, which were chosen based on the literature and the outcomes of our actual work, were lamp power (A), pH (B), and catalyst quantity (C). Except for pH, all input parameters should be set up at three levels (− 1, 0 + 1) at equally spaced intervals. To improve the RSM model, software called Design Expert-13 was utilized. The input parameters and their levels are shown in Table 1. There were twenty trials carried out in all. Table 2 shows the actual and anticipated results for the volume of H_2_ generation produced by the D-optimal design. Equation (1) illustrates the response surface that was estimated by the RSM model using the secondary polynomial model^38^.

1 $${\text{Y }} = \beta_{0} + \beta_{{\text{a}}} {\text{A }} + \beta_{{\text{b}}} {\text{B }} + \beta_{{\text{c}}} {\text{C }} + \beta_{{{\text{ab}}}} {\text{AB }} + \beta_{{{\text{ac}}}} {\text{AC }} + \beta_{{{\text{bc}}}} {\text{BC }} + \beta_{{{\text{aa}}}} {\text{A}}^{2} + \beta_{{{\text{bb}}}} {\text{B}}^{2} + \beta_{{{\text{cc}}}} {\text{C}}^{2}$$

where Y is the predicted output response, A, B, and C are independent factors, *β*_a_, *β*_b_ and *β*_c_ are the coefficients, *β*_0_ is the intercept constant term and *β*_aa_, *β*_bb_ and *β*_cc_ are the interactive coefficients.

**Table 1:** Range and levels used for the batch desorption study.

Factor	Name	Units	Type	Minimum	Maximum	Coded low	Coded high	Mean	Std. dev.
A	Lamp	W	Numeric	400.00	1200.00	− 1 ↔ 400.00	+ 1 ↔ 1200.00	800.00	343.36
B	pH		Numeric	4.00	10.00	− 1 ↔ 4.00	+ 1 ↔ 10.00	7.30	2.85
C	DOSE	mg	Numeric	600.00	800.00	− 1 ↔ 600.00	+ 1 ↔ 800.00	705.00	82.56

**Table 2:** Comparison between expected and actual results for several experimental runs.

Run	Independent factors			H_2_ production (µmol)	
	Lamp (W), A	pH, B	Dose (mg), C	Exp	Predicted
1	1200	10	700	6246.1	4943.41
2	800	10	600	1963.06	1863.6
3	400	10	600	446.15	801.64
4	400	6	600	446.15	433.59
5	400	4	700	446.15	598.03
6	800	4	600	2052.29	2139.27
7	400	4	700	446.15	598.03
8	800	4	600	2052.29	2139.27
9	800	10	700	2766.13	2185.94
10	400	10	800	1338.45	1009.32
11	1200	4	800	5353.8	5033.36
12	1200	4	800	5353.8	5033.36
13	1200	10	600	4372.27	4822.01
14	1200	10	800	3747.66	4225.96
15	400	10	700	446.15	1324.9
16	400	10	800	1338.45	1009.32
17	1200	6	700	4193.81	4731.02
18	1200	10	800	3747.66	4225.96
19	800	4	700	2141.52	2304.67
20	800	6	800	446.15	788.68

To optimize the three independent variables, a bespoke design with six axial points, eight factorial points, and six repetitions at the center point was needed. Five degrees of variation were applied to the chosen variables (− 1, 0, + 1). Equation (2) was used to compute the number of experiment runs.

2 $${\varvec{N}} = \, 2^{k} + \, 2.k + C = \, 2^{3} + \, 2.3 \, + \, 6 = 20$$

where *C* is the total number of experiments carried out at the center, *N* is the number of runs, and *k* is the number of variables to be examined. ANOVA was employed to do a statistical analysis of the final model. To look at the connections between the variables, surface contour plots were used.

## Results and discussion

### Catalyst characterization

#### FTIR

**Figure 1:**
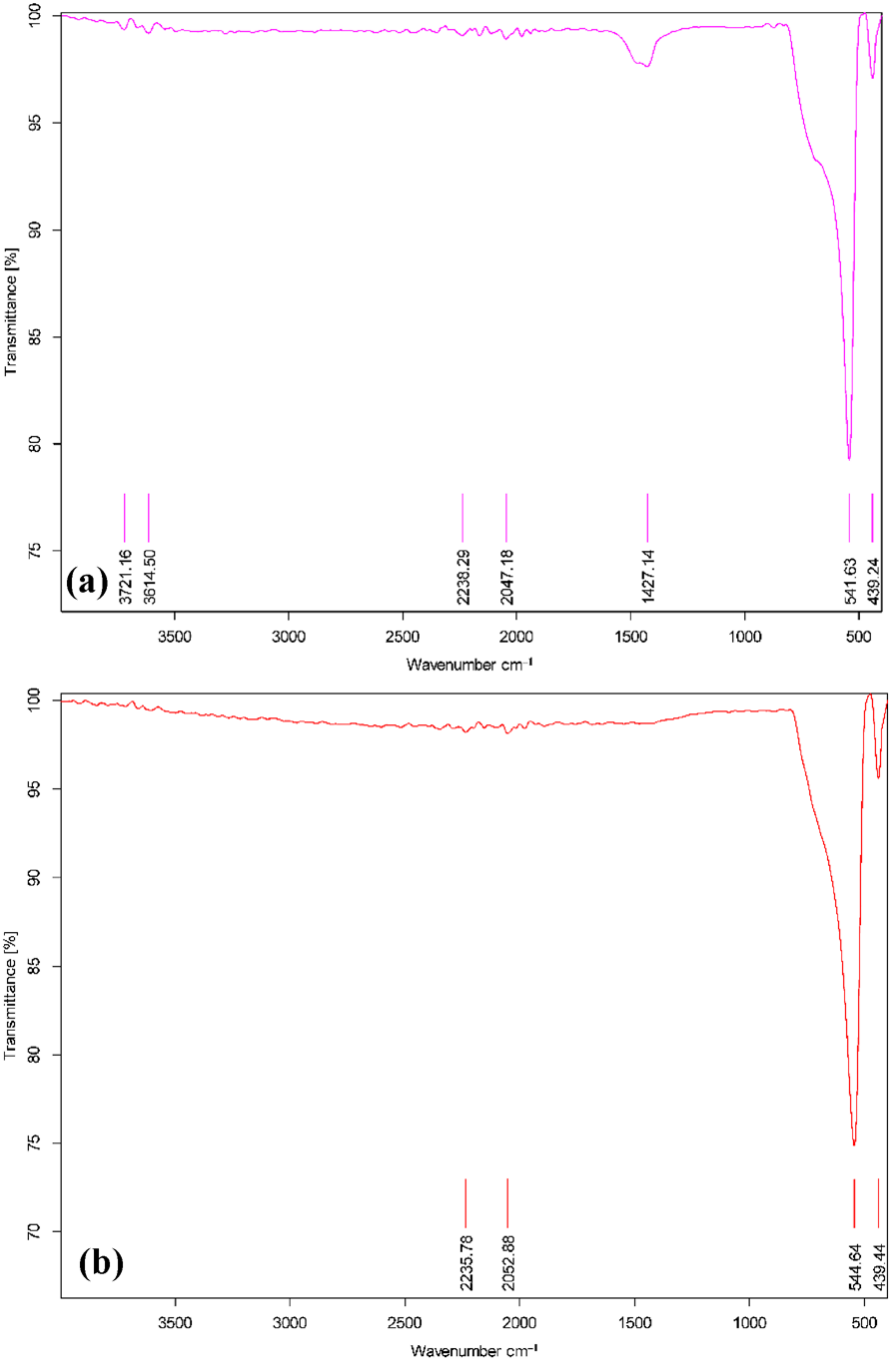
FTIR analysis of (**a**) CaTiO_3_ and (**b**) Ag-La-CaTiO_3_.

FT-IR analyses were performed for both Ag-La-CaTiO_3_ and CaTiO_3_ samples calcined at 850 °C (Fig. 1a,b). In the case of CaTiO_3_, the wide bands seen above 3614 and 3721 cm^−1^ were associated with the stretching vibration of the adsorbed O–H group and the superposition of the hydroxyl group’s vibration band. The hallmark peaks of the CaTiO_3_ bond were identified as the bands located at 541 and 544 cm^−1^. The Ti–O–Ti bond's bending mode was responsible for the absorption peak seen at 439 cm^−1^. The Ti–O stretching vibration and Ti–O–Ti bridging stretching mode were identified as absorption peaks at 544 and 439 cm^−1^, respectively^39^. This suggests the creation of a CaTiO_3_ perovskite-type structure and the presence of TiO octahedral. A band at 1427 cm^−1^ is also seen in the FTIR spectra. The FTIR band at 1400 cm^−1^ displays both symmetrical and asymmetrical vibrations between metal oxides, per the earlier study. As a result, it shows the bond vibration between the C and O of CO_3_^2−^ ions, which is the remaining interaction of the CaCO_3_ functional group^39–41^. Ag-La CaTiO_3_’s FT-IR spectra exhibit the same general pattern as pure CaTiO_3_. The outcomes demonstrated that CaTiO_3_ was successfully doped with Ag and La ions^40,41^. Therefore, significant structural confirmation is provided by the FT-IR spectra of the as-synthesized CaTiO_3_ and Ag-La-CaTiO_3_.

#### Scanning electron microscope (SEM)

**Figure 2:**
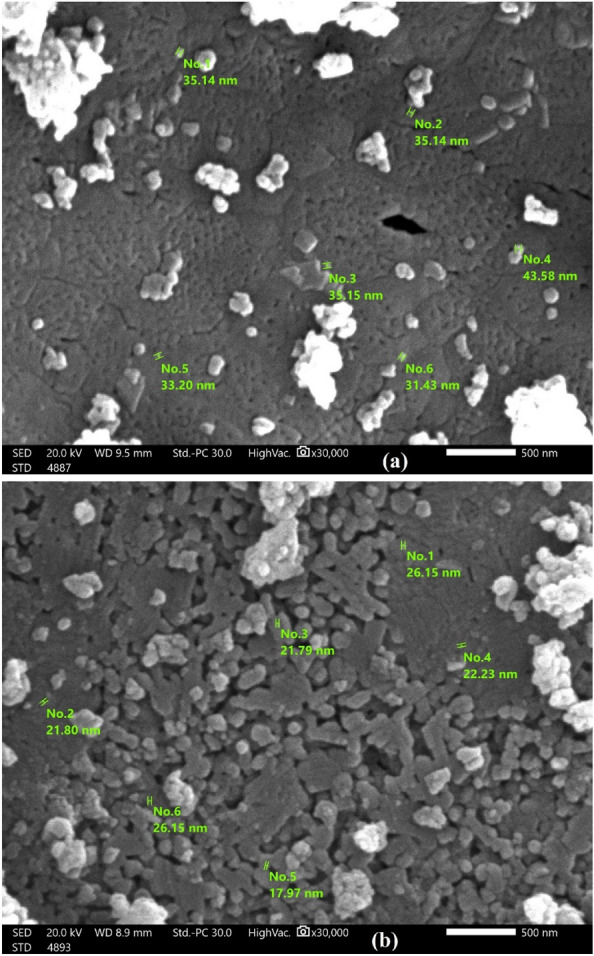
SEM image analysis of (**a**) CaTiO_3_ and (**b**) Ag-La-CaTiO_3_.

The field emission SEM device was applied at a magnifying power of 30,000 × to examine the surface morphology of CaTiO_3_ and Ag-La-CaTiO_3_. Images of synthesized CaTiO_3_ and Ag-La CaTiO3 are presented in Fig. 2a,b. The pictures illustrate that the substance has an uneven morphology. Comparing Fig. 2a of CaTiO_3_ with Fig. 2b of Ag–La-CaTiO_3_ shows a significant influence on the morphologies of Ag–La codoping CaTiO_3_ sample. After Ag–La codoping, the grain size often changes, becoming smaller. The photocatalytic activity benefits from the tiny particle^10,39^. This result is in line with findings reported in the literature, which contend that the highly photocatalytic activities are caused by a decrease in the migration distance of photogenerated electrons and holes to reach the reaction site on the surface as a result of particle size reduction^10,39^.

#### EDX analysis

**Figure 3:**
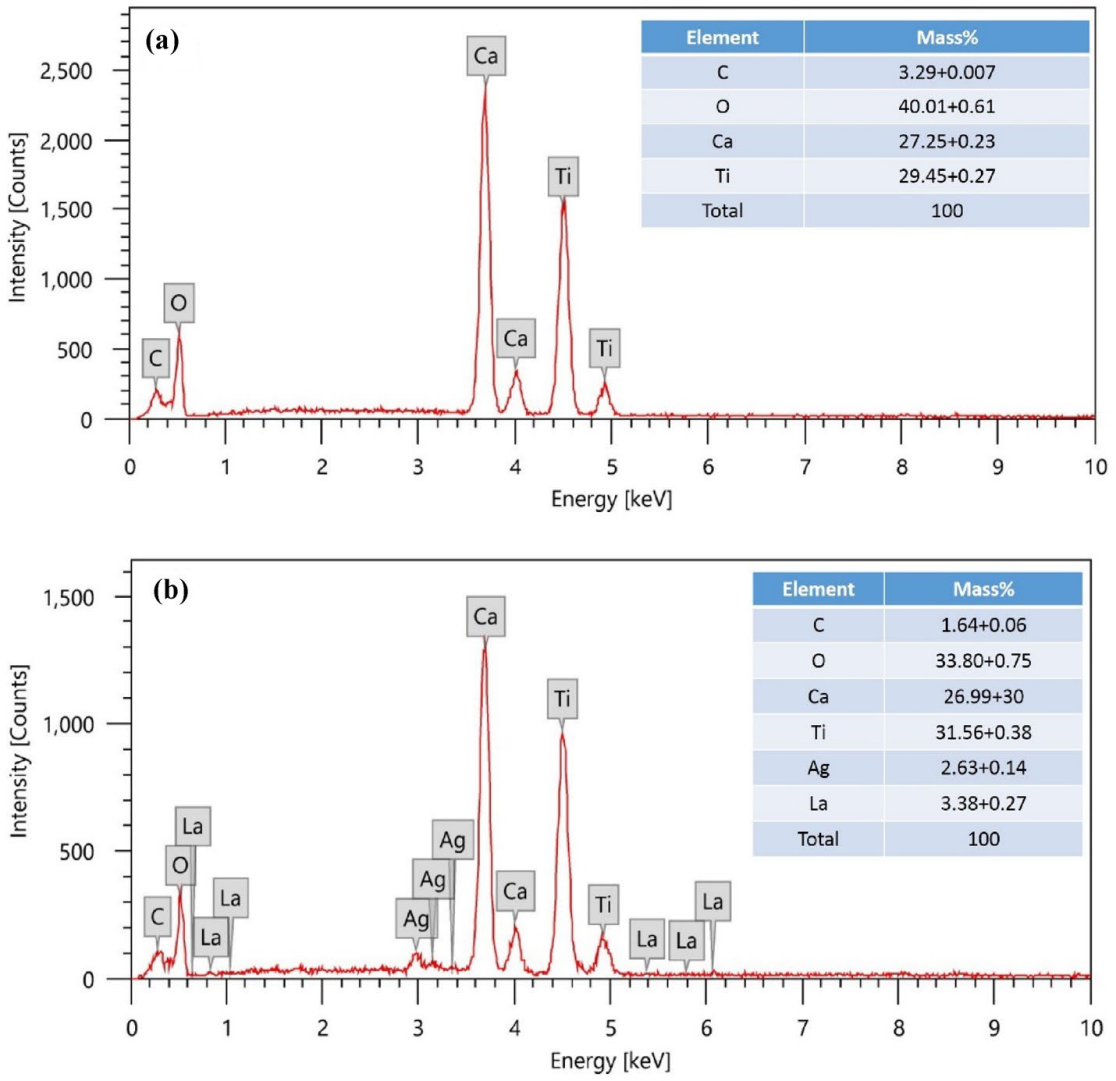
(**a**) EDX analysis of CaTiO_3_ calcined at 850 °C and (**b**) EDX analysis of AG-La doped CaTiO_3_ calcined at 850 °C.

Only the elemental peaks for Ca, Ti, O, C, Ag, and La were visible in the recorded EDX spectrum in Fig. 3, confirming the existence of these critical elements in the as-synthesized CaTiO_3_ (Fig. 3a) and Ag, La doped CaTiO_3_ (Fig. 3b). The findings showed that the sole components of CaTiO_3_ and Ag, La doped CaTiO_3_ were their respective atoms, with no impurities^39^.

#### UV–Vis diffuse reflection spectra

**Figure 4:**
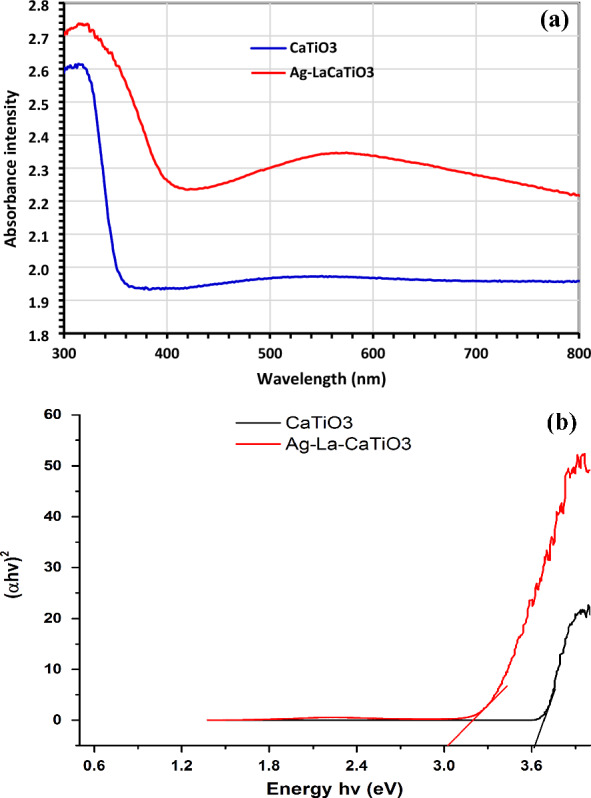
(**a**) DRS analysis of CaTiO_3_ and Ag-La-CaTiO_3_, (**b**) Tauc plot of CaTiO_3_ and Ag-La-CaTiO_3_.

Figure 4a displays the room-temperature diffuse reflection spectra of the photocatalysts. Through the Kubelka–Munk technique, the absorbance intensity was converted from the reflectivity spectrum. These photocatalysts' absorbance spectra begin to shift towards longer wavelengths at which point Ag^+^ and La^3+^ replace Ca^2+^. The development of the redshift may be explained by the charge-transfer transition between the electrons in Ag ions at 4d5s and the O 2p + Ti 3d hybrid orbital. In the visible light range, two forms of absorption are produced for the Ag-La codoped CaTiO_3_ samples: a wide absorption that extends into the visible region and a significant absorption in the UV region shorter than 350 nm. Along with the primary CaTiO_3_ peak at 350 nm, Ag-La-CaTiO_3_ adds a band at < 560 nm in the visible range between 400 and 800 nm. The transition of Ag 4d5s electrons to the conduction band (CB) is responsible for the absorption band at ~ 560 nm (> 2.21 eV). Equation (3) was used to compute the energy bandgap (Eg) using Tauc's relation^42^.

3 $$\alpha hv = A(hv - E_{g} )^{n}$$

In this case, “*n*” is a constant equal to 2 for an indirect transition and 1/2 for a direct transition, “*A*” is a constant, and “*α*” is the absorption coefficient. Figure 4b shows the (*αhυ*)^2^ values vs photon energy (*hυ*). It was discovered that the optical direct energy bandgap (*Eg*) values for Ag–La–CaTiO_3_ and CaTiO_3_ were 3.002 and 3.618 eV, respectively. As stated by Zhang et al.^10^, it was discovered that the *Eg* value decreased following doping Ag and La concentration.

#### XRD analysis

**Figure 5:**
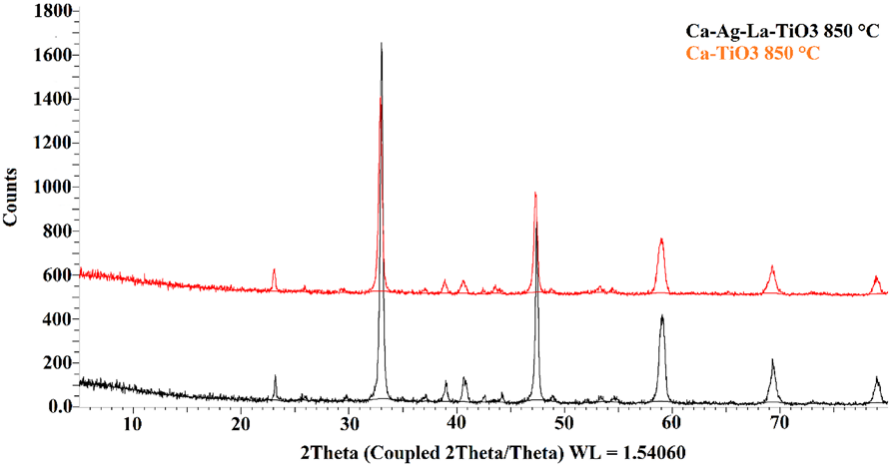
XRD analysis of (**a**) CaTiO_3_, and (**b**) Ag-La-CaTiO_3_.

Figure 5 displays the XRD patterns of both pure CaTiO_3_ and Ag-La codoped CaTiO_3_. Only the CaTiO_3_ orthorhombic host lattice phase may be used to index the pattern; no contaminants are found. Ag-La-CaTiO_3_ powder X-ray diffraction patterns are very similar to one another. Upon further examination, it was evident from Fig. 5 that the peak locations of the (110) diffraction peaks in the 2*θ* 33.012° range are somewhat moved to higher angles with Ca_0.94_Ag_0.03_La_0.03_. The shift suggests that the CaTiO_3_ lattice has at least some homogeneous Ag-La doping. In comparison to Ti^4+^ (0.68 Å), which is located in the location of *B* sites in perovskite structures, the ionic radii of La^3+^ (1.18 Å) and Ag^+^ (1.15 Å) are closer to the Ca^2+^ ion (1.00 Å). There should be a significant change if Ag^+^ and La^3+^ take the role of Ti^4+^. Ca ions occupying A sites in perovskite structures are thus thought to have been replaced in the bulk by Ag and La ions, as shown by the slight changes to higher angles seen in the diffraction patterns of Ca_0.94_Ag_0.03_La_0.03_. These findings are consistent with those of Zhang et al.^10^, who synthesized the identical chemical and discovered 110 diffraction peaks at 2*θ* 33.1°.

#### XPS analysis

**Figure 6:**
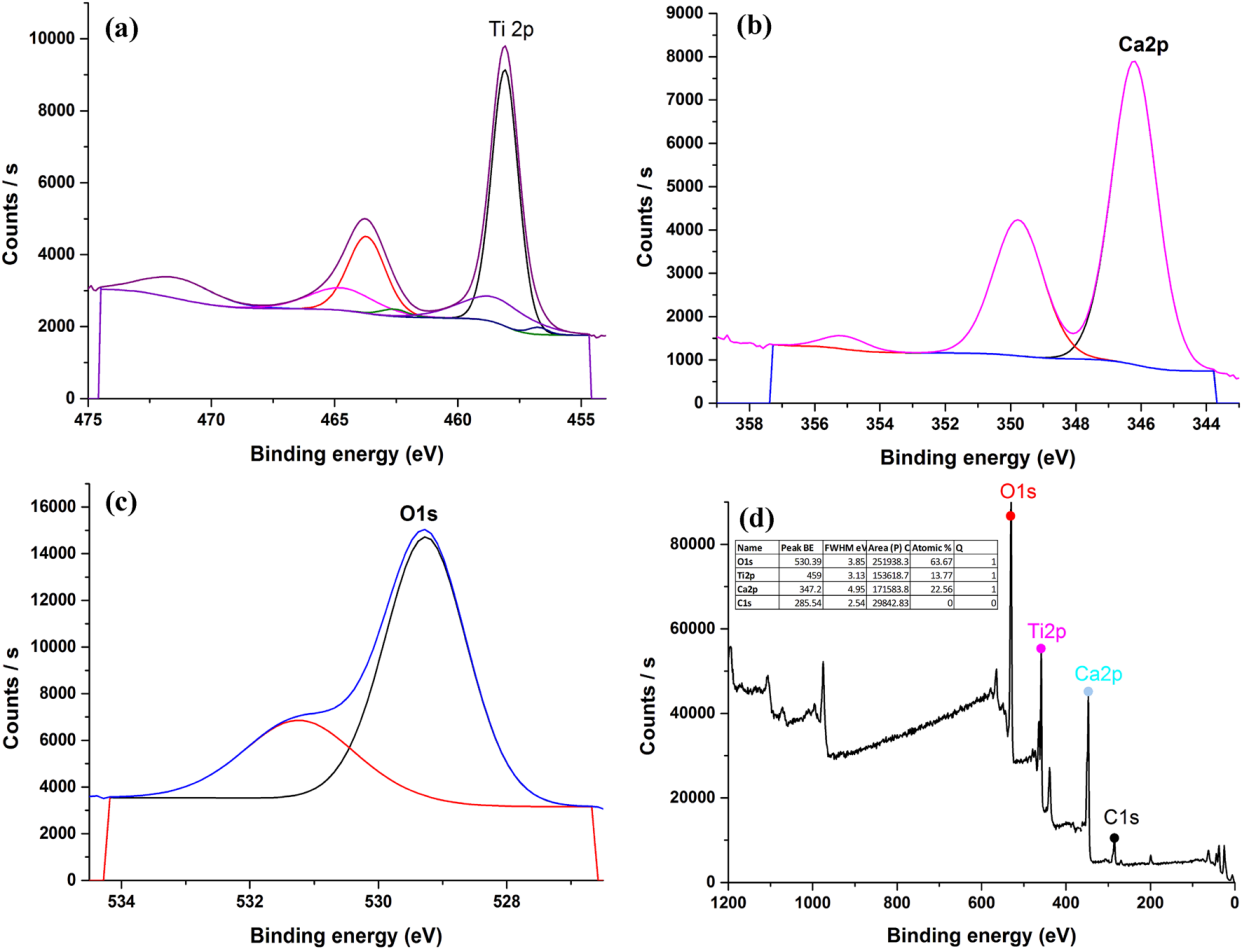
XPS analysis of CaTiO_3_ (**a**) Ti, (**b**) Ca, (**c**) O, (**d**) Survey.

**Figure 7:**
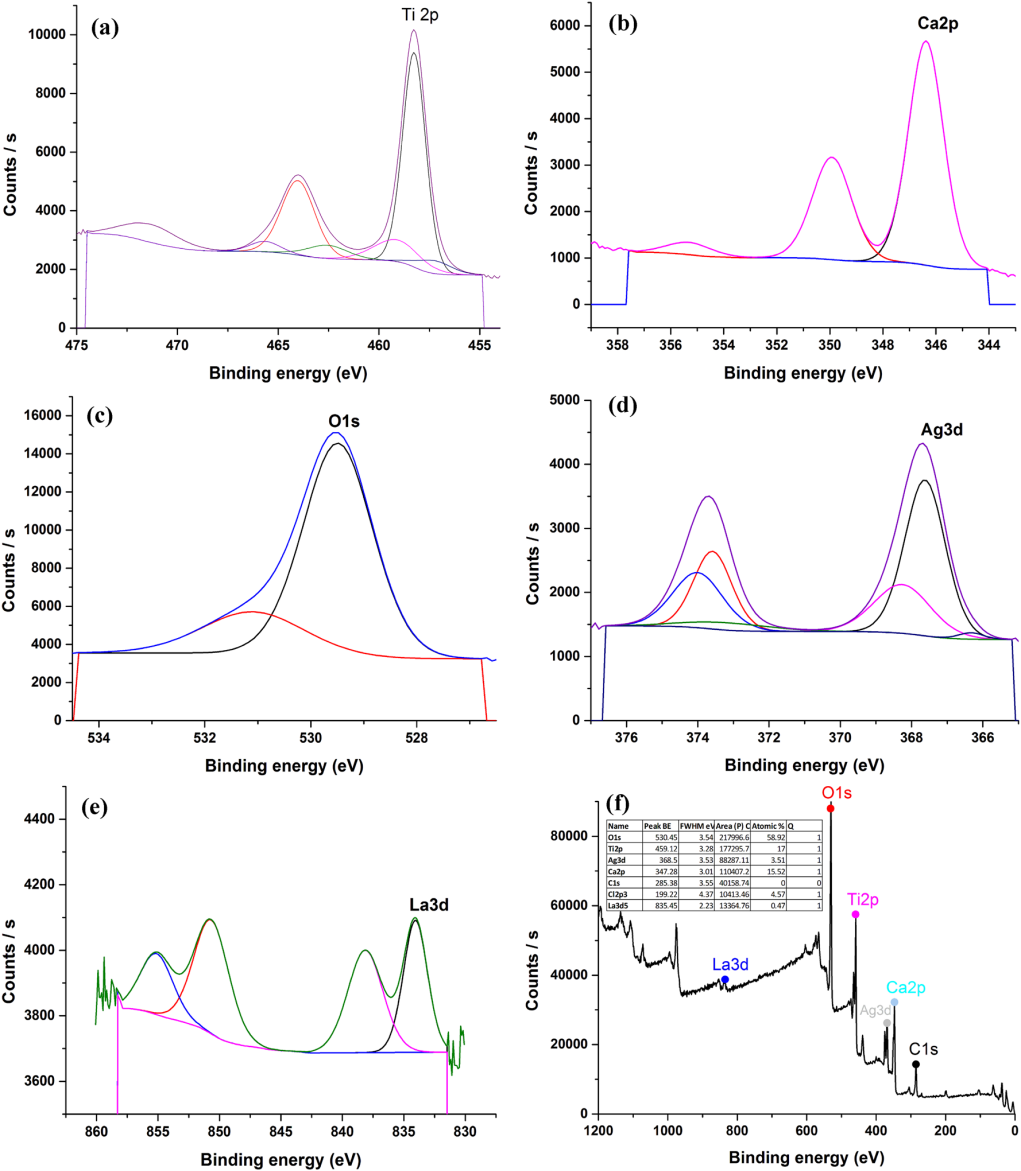
XPS analysis of Ag–La codoped CaTiO_3_ (**a**) Ti, (**b**) Ca, (**c**) O, (**d**) Ag, (**e**) La, (**f**) Survey.

The XPS survey spectra of CaTiO_3_ and Ag-La codoped CaTiO_3_, which have surface elements of Ti, O, Ca, Ag, and La, are displayed in Figs. 6 and 7. XPS measurements were performed on the prepared samples at 850 °C to further establish the elemental composition and chemical state of CaTiO_3_ and Ag-La codoped CaTiO_3_. XPS spectra are shown in Fig. 6. By the results of the EDX, the survey spectrum shows that the primary components on the surface of CaTiO_3_ are Ca, Ti, and O, whereas the surface of Ag-La-CaTiO_3_ shows Ag, La, Ca, Ti, and O. C 1s at 285.5 eV corresponds to the adventitious carbon is used to calibrate the peak positions of all the elements.

The chemical state of Ca^2+^ is represented by two peaks in the high-resolution XPS spectra of Ca (Fig. 6) that are situated at about 346.21 and 349.77 eV, respectively. These peaks correspond to the Ca 2p_3/2_ and Ca 2p_1/2_. These measurements are consistent with those published for CaTiO_3_, and they indicate the existence of CaTiO_3_ (346.21 eV) along with some CaCO_3_ (349.77 eV). The existence of Ti^4+^ in the CaTiO_3_ photocatalyst is confirmed by the two primary peaks in the Ti XPS spectra, which are located at 458.09 and 463.8 eV and correspond to the Ti 2p_3/2_ and Ti 2p_1/2_, respectively. The two peaks at 529.26 in Fig. 6 may be fitted to the O 1s profile, and the minor peaks at 531.1 eV can be attributed to the chemisorbed oxygen brought on by the surface hydroxyl (OH)^43^.

However, Fig. 7 displays the Ag-La codoped CaTiO_3_ XPS spectrum. Two peaks in the high-resolution XPS spectra of Ca (Fig. 7) indicate the chemical state of Ca^2+^ and are situated at about 346.37 and 349.92 eV, respectively. These peaks correspond to the Ca 2p_3/2_ and Ca 2p_1/2_. These findings are consistent with previously published data and imply the existence of CaTiO_3_ (346.37 eV) along with some CaCO_3_ (349.92 eV)^43^. The existence of Ti^4+^ in Ag-La-CaTiO_3_ photocatalyst is confirmed by the XPS spectra of Ti, which exhibits two major peaks at 458.26 and 464.02 eV, respectively, that correspond to the Ti 2p_3/2_ and Ti 2p_1/2_^10^. Two peaks in Fig. 7’s O 1s profile, attributed to Ca–O or Ti–O, may be fitted to the profile. The minor peaks at 531.1 eV can be attributed to chemisorbed oxygen, which is created by the surface hydroxyl (OH)^43^. Furthermore, in Ag–La codoped CaTiO_3_, the binding energies of Ag 3d_3/2_ and Ag 3d_5/2_ are 367.62 and 373.59 eV, respectively (Fig. 7). La_2_O_3_ is represented by the peak of La 3d5, which is 834.05 eV, while La^3+^ is represented by the XPS peak in Fig. 7, which is positioned at around 838.14 eV.

#### BET

The BET technique was used to analyze the surface area of CaTiO_3_ and Ag-La codoped CaTiO_3_ (Table 3). With a specific surface area of 14.75 m^2^/g, CaTiO_3_ has a smaller specific surface area than Ag–La codoped CaTiO_3_, which has a specific surface area of 15.43 m^2^/g, based on the multipoint BET equation. The table displays the specific surface area (BET) and the relationship between the doping quantity and the photocatalytic activity of both Ag-La codoped CaTiO_3_ and un-doped CaTiO_3_ under visible light. The table shows that after codoped Ag–La, the specific surface areas increase, which is consistent with the SEM result^10^. At 3 mol% doping, the photocatalytic activity rose. It is commonly recognized that there is an ideal value for the doped ion concentration. Because there are not as many charge carrier capture traps in the semiconductor when the doped concentration is below the ideal doped concentration, photocatalytic activity increased as the doped concentration rose. Because of the limited solubility of doped ions in CaTiO_3_, a higher doped concentration may result in doped ion enrichment on the catalyst surface, which lowers photocatalytic activity. On the other hand, the recombination rate will increase and the average distance between capture traps will decrease when the doped concentration is less than the ideal doped concentration. Ag–La codoped CaTiO_3_ powder exhibits significantly greater photocatalytic activity for hydrogen evolution under visible light in comparison to pure CaTiO_3_ powder.

**Table 3:** Surface area, porosity analysis and H_2_ evolution of CaTiO_3_ and Ca_0.94_Ag_0.03_La_0.03_TiO_3_.

Sample	Surface area (m^2^/g)	The mean diameter of pores (nm)	The total volume of pores (cm^3^/g)	H_2_ production (μmol)
CaTiO_3_	14.75	10.881	0.0401	0.0
Ca_0.94_Ag_0.03_La_0.03_TiO_3_	15.43	6.611	0.0255	6246.09

#### TGA

**Figure 8:**
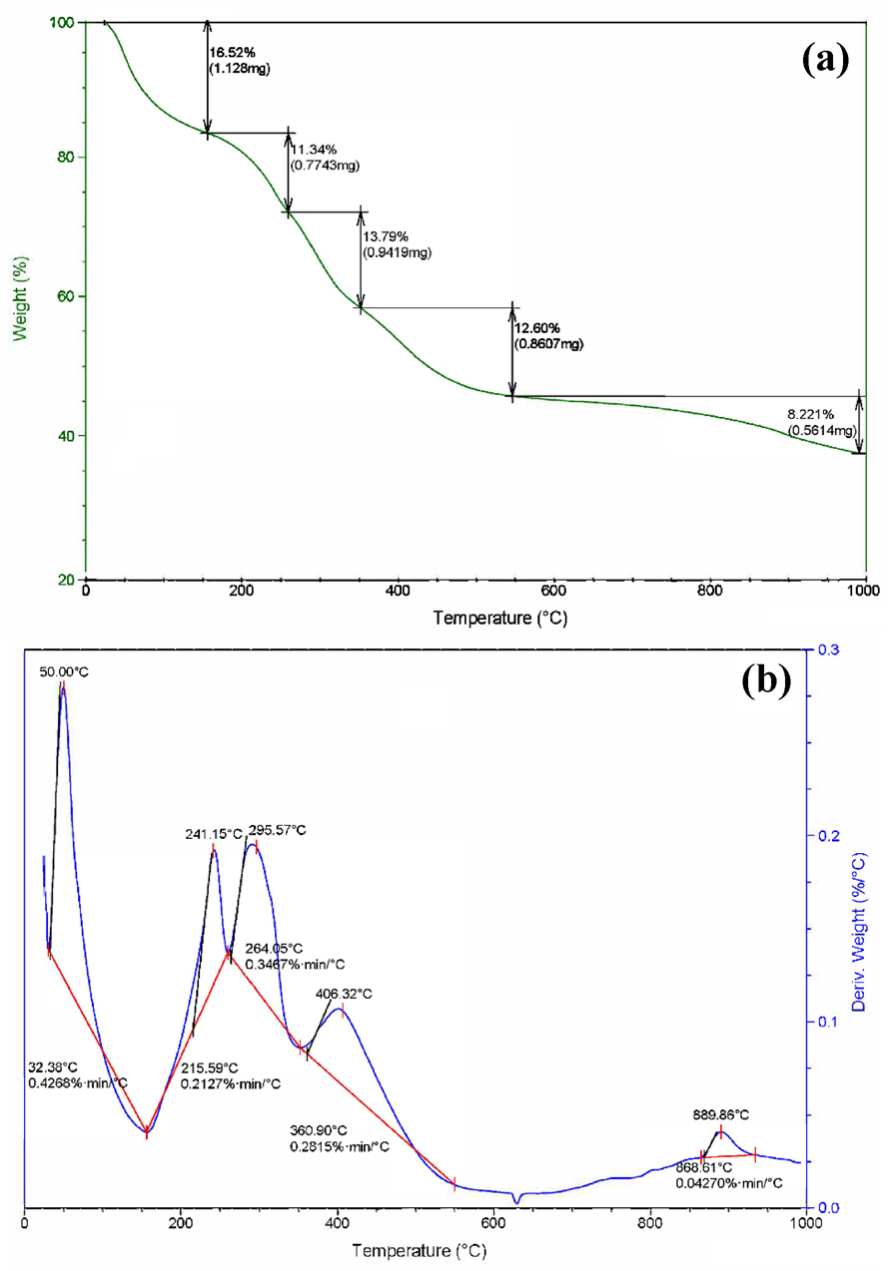
(**a**) TGA analysis of CaTiO_3_, and (**b**) DTA analysis of CaTiO_3_.

Thermogravimetric (TGA) and differential thermal analysis (DTA) were applied to examine the thermal stability of CaTiO_3_ produced at 120 °C. The samples were heated at a rate of 10 °C min^−1^ under a nitrogen environment, from 25 to 1000 °C (Fig. 8a,b). The initial peak of weight loss, which happened between 50 and 150 °C, was linked to a decrease in water content. This was followed by four weight losses in ranges between 150–250, 250–350, 350–550, and 550–1000 °C representing different thermal decompositions or transformations. On the other hand, DTA showed five different losses at 50, 241.15, 295.57, 406.57, and 889.86 °C. The weight loss peaks between 200 and 450 °C may correspond to the decomposition or removal of organic or carbon-containing species present in the sample. The weight loss peak after 550 °C might be associated with the decomposition or phase transition of CaTiO_3_ itself. Finally, the weight loss peak at 1000 °C could be related to the complete decomposition or phase transformation of any remaining components in the sample. Temperatures above 500 °C are necessary for titanate production, while temperatures up to about 800 °C cause the compound to lose weight. As a result, the titanate generation and carbonate breakdown are indicated by the DTA exothermic peak, which is located between 400 and 890 °C.

### Water splitting by photocatalytic activity

#### Effect of pH

**Figure 9:**
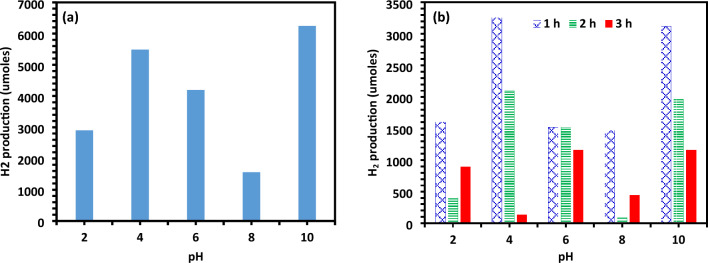
(**a**) Photo catalytic of total H_2_ generation for varying pH (2–10); (**b**) Photo catalytic H_2_ generation (µmol) during time interval at different pH (2–10).

Several batches of experiments were conducted to examine the impact of changing pH levels for optimal photocatalytic activity, with pH levels ranging from 2 to 10 (Fig. 9a,b). The generation of hydrogen utilizing Ag-La-CaTiO_3_ at different pH levels is shown in Fig. 9a under the light of a 1200 W metal halide lamp. After three hours in the dark, no visible hydrogen generation was seen. Nonetheless, under visible light illumination, clear H_2_ generation was detected in the Ag-La-CaTiO_3_-amended system, suggesting that photo-assisted water splitting is the primary source of H_2_ generation. The highest H_2_ generation was attained at pH 4 and 10 with H_2_ yields of 5487.6 and 6246.09 µmol, respectively. The water-splitting reaction and high hydrogen (H_2_) yield using Ag-La-CaTio_3_ can occur at different pH values due to the variation in the redox potential and surface charge of the catalyst material. The solution pH affects the concentration of H^+^ and OH^−^ ions, which, in turn, influences the reaction kinetics and surface reactions.

At pH 4, the solution is acidic, meaning there is a higher concentration of H^+^ ions (protons). The water-splitting reaction can be represented as in Eqs. (4) and (5).

4 $$2{\text{H}}_{2} {\text{O}}\;({\text{L}}) \, \to {\text{ O}}_{2} \;({\text{g}}) \, + \, 4{\text{H}}^{ + } \;({\text{aq}}) \, + \, 4{\text{e}}^{-}$$

5 $${\text{H}}^{ + } \;({\text{aq}}) \, + \, 4{\text{e}}^{-} \to \, 2{\text{H}}_{2} ({\text{g}})$$

In acidic conditions (pH 4), there is already an abundance of H^+^ ions available in the solution. The presence of Ag-La-CaTiO_3_ as a catalyst can facilitate the electron transfer (4e^−^) to the H^+^ ions, supporting the production of hydrogen gas (H_2_) with high yield.

At pH 10, the solution is basic, meaning there is a higher concentration of OH^−^ ions (hydroxide ions). The water-splitting reaction can be represented in Eqs. (6) and (7).

6 $$2{\text{H}}_{2} {\text{O}}\;({\text{L}}) \, + \, 2{\text{e}}^{-} \to {\text{ H}}_{2} \;({\text{g}}) \, + \, 2{\text{OH}}^{-} \;({\text{aq}})$$

7 $${\text{OH}}^{-} \;({\text{aq}}) \, \to \, 2{\text{H}}_{2} {\text{O}}\;({\text{L}}) \, + {\text{ O}}_{2} \;({\text{g}}) \, + \, 4{\text{e}}^{-}$$

In basic conditions (pH 10), there is an abundance of OH^−^ ions available in the solution. The Ag-La-CaTiO_3_ catalyst can facilitate the transfer of electrons (2e^−^) to the OH^−^ ions, promoting the production of hydrogen gas (H_2_) with high yield.

Additionally, a higher pH can result in a concentration of hydroxyl ions that can combine with holes to create hydroxyl radicals, which will improve the rate of photocatalysis as shown by Eqs. (8–11).

8 $${\text{Ag-La-CaTiO}}_{3} + hv \to {\text{ Ag - La - CaTO}}_{3} {\text{H}}^{ + } + {\text{e}}^{-}$$

9 $${\text{OH}}^{-} + {\text{ h}}^{ + } \to {\text{ OH}}^{\cdot}$$

10 $${\text{OH}}^{-} + hv \to {\text{OH}}^{\cdot} + {\text{ e}}_{{{\text{aq}}}}^{-}$$

11 $${\text{e}}_{{{\text{aq}}}}^{-} + {\text{ O}}_{2} \to {\text{ O}}_{2}^{\cdot}$$

The water splitting as indicated in Fig. 9a,b occurs in other pH is may be due to direct photolysis of the solution system where hydroxyl radicals (OH.) and hydrated electrons (e_aq_^−^) can be formed when water is irradiated with high energy Vis light.

#### Effect of catalyst

**Figure 10:**
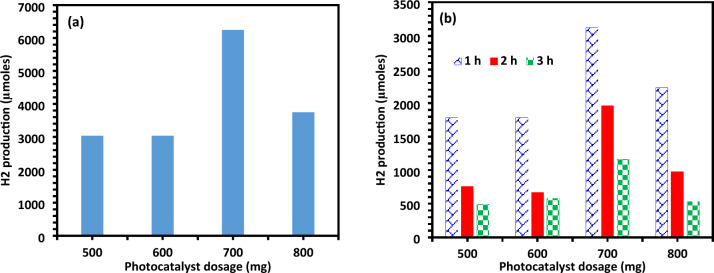
(**a**) Photo catalytic of total H_2_ generation for varying catalytic loading of Ag-La-CaTiO_3_, (**b**) Photo catalytic H_2_ generation (µmol) during time interval for Ag-La-CaTiO_3_.

Several batches of experiments were conducted to examine the impact of catalyst loading for optimal photocatalytic activity. The amount of catalyst used varied between 500 and 800 mg. A comparison was made between the photocatalytic activity of the photocatalyst and the photocatalytic yield of hydrogen to determine the impact of catalyst loading on photocatalytic water splitting. It was observed that H_2_ production increased and reached an optimal point with an increase in catalyst loading (700 mg) when the amount of photocatalyst (Ag-La-CaTiO_3_) was varied to 500, 600, 700, and 800 mg while maintaining identical operating parameters. It can be shown from Fig. 10a that the hydrogen yield increased as the catalyst loading increased. Catalyst loading varied from 500 to 600 mg, resulting in about the same yield of 3030 µmol of hydrogen production; when it was raised from 500 to 700 mg, the yield rose by 6300 µmol. However, the hydrogen production significantly decreased to 3700 µmol when the catalyst loading was increased from 700 to 800 mg. The optimal catalyst loading, as indicated by the hydrogen production curves plotted in Fig. 10a, is 700 mg. This was further supported by the bar graph in Fig. 10b, which showed that at the first hour, the photocatalytic activity for a 700 mg photocatalyst was 3120 µmol g^−1^ h^−1^, suggesting that 700 mg was the ideal loading of the catalyst.

#### Effect of light intensity

**Figure 11:**
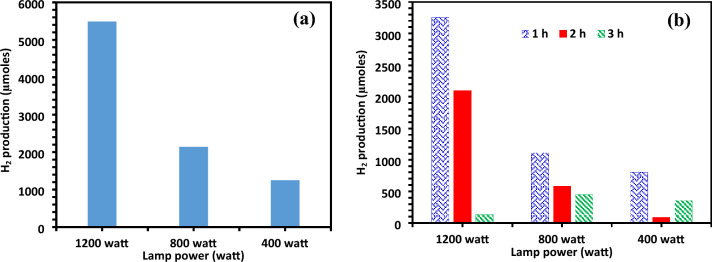
(**a**) Photo catalytic of total hydrogen generation for varying light intensities (200–1200 W). (**b**) Photocatalytic hydrogen generation (µmol) during time intervals at various light intensities (200–1200 W).

Several batches of experiments were conducted to examine the impact of changing light intensities for optimal photocatalytic activity. The intensities were varied within the 400–1200 W range (Fig. 11a,b). The highest H_2_ production was attained at the illumination of 1200 W with a H_2_ yield of 5487.64 µmol after 3 h with a catalyst dose of 700 mg and pH 4. The water-splitting reaction rate, such as the generation of hydrogen and oxygen, is directly influenced by the intensity of the incident light. Higher light intensity generally leads to increased reaction rates, as more photons are available to provide energy for the reactions involved in water splitting.

#### Catalyst reusability

**Figure 12:**
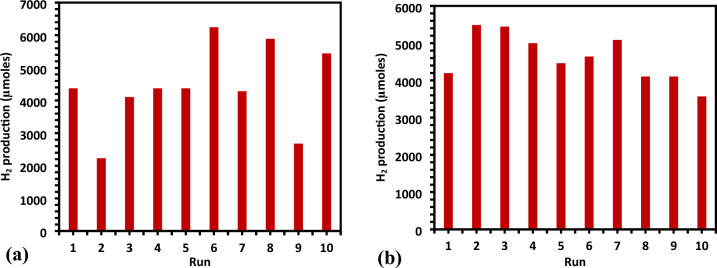
(**a**) Recyclability of photocatalytic activities of Ag–La-CaTiO_3_ over a span of 10 cycles (30 h) at pH 10, (**b**) recyclability of photocatalytic activities of Ag–La-CaTiO_3_ over a span of 10 cycles (30 h) at pH 4.

**Figure 13:**
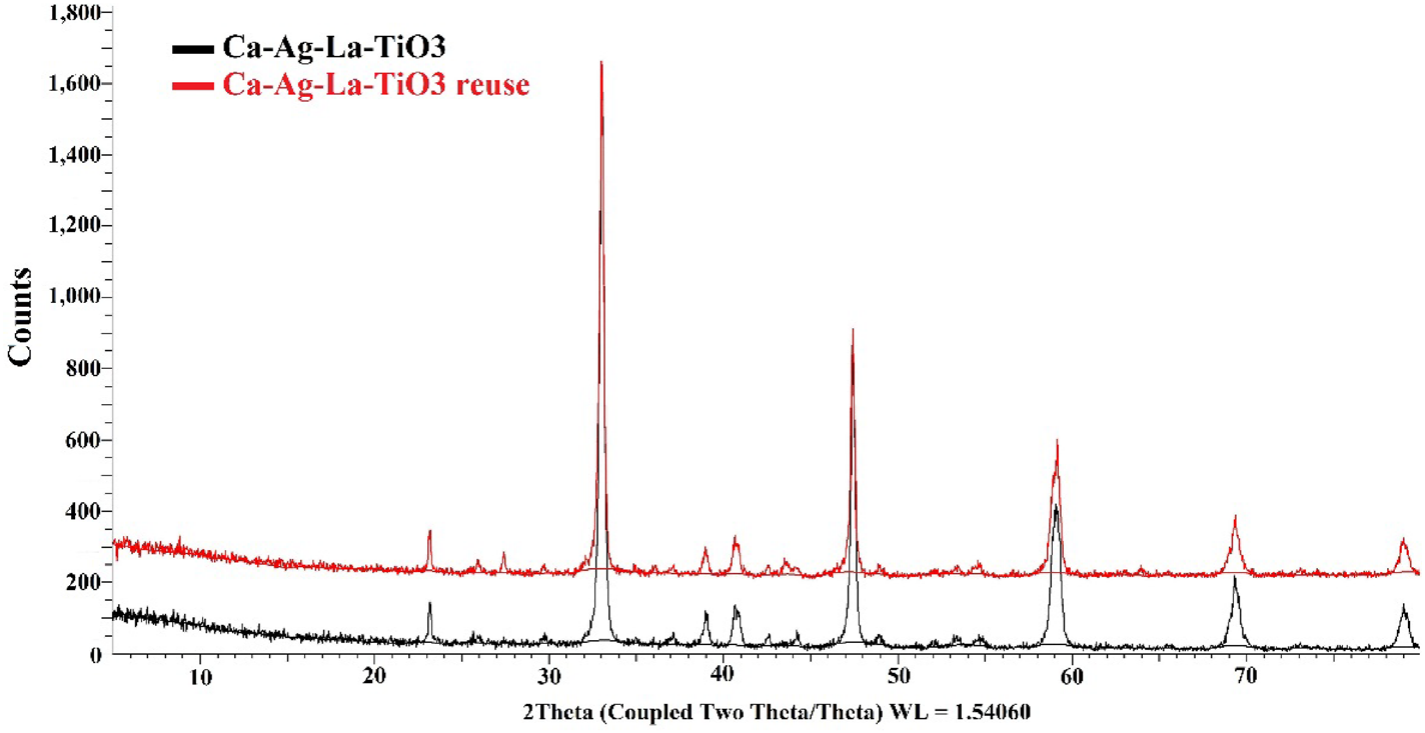
XRD pattern of fresh and reused Ag–La-CaTiO_3_ photocatalyst.

The photo catalyst's recyclability was investigated over the course of ten cycles, each lasting three hours. It is obvious from Fig. 12a,b that the catalyst deactivated over time at two distinct pH values of 4 and 10. The fresh synthesized catalyst Ag-La-CaTiO_3_ was calcined and used for photocatalysis processes for water splitting and 6246.096 µmol of hydrogen was generated during the first 3 h of operation. Before each run of the recyclability, the solution was purged for 15 min. with N_2_ gas and the pH was adjusted. After 30 h of 10 runs, the average yield of hydrogen was 43,099.04 ± 1268.93 and 4608.73 ± 635.16 µmol for pH 10 and 4, respectively. Catalytic activity decreased from 6246 to 2676.9 µmol g^−1^ h^−1^ and from 5487.64 to 3569.2 µmol g^−1^ h^−1^ for pH 10 and pH 4, respectively (Fig. 12a,b). This indicates that the photocatalyst has a high rate of reusability since the synthesized catalyst may be utilized again even after ten runs. However, as can be seen from Fig. 12b, photocatalytic activity gradually decreased over time due to a decrease in the photocatalyst's active sites brought on by the deposition of intermediate oxidized products. Nevertheless, photocatalytic activity can be increased by calcining Ag-La-CaTiO_3_. With a catalytic average activity of 43,099.04 and 4608.73 µmol g^−1^ h^−1^ for pH 10 and pH 4, respectively, the catalyst may be recycled at least ten times. The cost of the catalyst's materials and synthesis (including drying and calcination) is around $10. Figure 13 displays the Ag-La codoped CaTiO_3_ XRD patterns for both fresh and recycled samples. Only the CaTiO_3_ orthorhombic host lattice phase may be used to index the pattern; no contaminants are found. Both newly made and previously used Ag-La-CaTiO_3_ powders have X-ray diffraction patterns that are strikingly comparable.

Table 4 shows the comparison of this work with other H_2_ production in the literatures. The H_2_ production in this study is higher (6246.09 μmol) than that obtained by different researchers^45–48^.

**Table 4:** Comparison of Ag-La-CaTiO_3_ with other photocatalysts in the H_2_ production application.

Photocatalyst	Light Source	Working conditions	Efficiency (μmol h^−1^ g^−1^)	References
Ag-La-CaTiO_3_	1200 W metal halide lamp	700 mg, pH 10, 3 h with no sacrificing agent	6246.09	This study
SrTiO_3_@Mo_2_C	300 W Xe lamp, 313 nm	25 mg catalyst, 90 mL of H_2_O/(10 vol%) TEOA	7900	^44^
AueAl/SrTiO_3_	150 W Xe lamp, simulated sunlight	2.5 mg catalyst, 1000 mL of H_2_O/30% isopropyl alcohol	347	^45^
Pt/NeTiO_2_/110T-SrTiO_3_	310 W/m^2^ Xe lamp	50 mL solution containing 25% v/v methanol	3873	^46^
SrTiO_3_-TiO_2_	300 W Xe lamp, > 400 nm	50 mg catalyst, 150 mL of DI H_2_O/H_2_PtCl_6_ solution (0.73 mg/mL)	3400	^47^
Ag(_3_) SrTiO_3_/g-CN	300 W Xe lamp, 420 nm	50 mg catalyst, 100 mL of H_2_O/(0.35 mol L^−1^ Na_2_S·9H_2_O and 0.25 mol L^−1^ Na_2_SO_3_)	645.62	^48^

### Response surface methodology

#### Model equation

Equations (12) and (13), which represent the coded and uncoded values of the model equation developed using the surface response approach, were provided. The formula for the electrolysis duration, electrode voltage, and catalyst quantity was written as A, B, and C, respectively.

12 $${\text{H}}_{{2}} = {1283}.{12 } + { 2232}.0{\text{5A }} - { 59}.{\text{37B }} - { 175}.{\text{56C }} - { 422}.{\text{8AB }} - { 2}00.{\text{93AC }} + { 78}.{\text{47BC }} + { 948}.{\text{27A}}^{{2}} + { 962}.{\text{18B}}^{{2}} - { 419}.{\text{42C}}^{{2}}$$

13 $${\text{H}}_{{2}} = -{ 16383}.{3 } + { 2}.0{8}0{\text{5Lamp }} - { 1417}.{\text{75pH }} - { 59}.{\text{15Lamp }} \times {\text{ pH }} - \, 0.00{5}0{\text{2Lamp }} \times {\text{ dose }} + \, 0.{\text{261546pH }} \times {\text{ dose }} + \, 0.0{\text{5926Lamp}}^{{2}} + 106.90{\text{pH}}^{{2}} - \, 0.0{\text{4194dose}}^{{2}}$$

Examining the aforementioned equations for the efficiency of hydrogen generation, the positive value of the “A” coefficient (2232.05) indicated that raising the lamp intensities from 400 to 1200 W was the most effective way to increase the volume of hydrogen. The model with a value of 13.31F and the model parameters A (Lamp), B (pH), AB, B2, and C2 were shown to be significant (p < 0.05) based on the data in Table 5.

**Table 5:** ANOVA for D-optimal design and a summary of model fit.

Source	Sum of squares	df	Mean square	F-value	p-value	Significant	PRESS	− 2 Log likelihood	BIC	AICc
Model	6.34E+07	9	7.04E+06	13.31	0.0002	Significant	–	–	–	–
A-Lamp	4.70E+07	1	4.70E+07	88.86	< 0.0001	–	–	–	–	–
B-pH	49,780.51	1	49,780.51	0.0941	0.7654	–	–	–	–	–
C-DOSE	2.70E+05	1	2.70E+05	0.5105	0.4912	–	–	–	–	–
AB	1.51E+06	1	1.51E+06	2.85	0.1223	–	–	–	–	–
AC	2.71E+05	1	2.71E+05	0.5122	0.4906	–	–	–	–	–
BC	51,221.38	1	51,221.38	0.0968	0.7621	–	–	–	–	–
A^2^	2.94E+06	1	2.94E+06	5.56	0.0400	–	–	–	–	–
B^2^	1.78E+06	1	1.78E+06	3.36	0.0966	–	–	–	–	–
C^2^	6.68E+05	1	6.68E+05	1.26	0.2874	–	–	–	–	–
Residual	5.29E+06	10	5.29E+05	–	–	–	–	–	–	–
Lack of fit	5.29E+06	5	1.06E+06	–	–	–	–	–	–	–
Pure error	0	5	0	–	–	–	–	–	–	–
Cor total	6.87E+07	19					–	–	–	–
SD	727.44	–	–	–	–	–	–	–	–	–
Mean	2467.21	–	–	–	–	–	–	–	–	–
C.V.%	29.48	–	–	–	–	–	–	–	–	–
R^2^	0.9230	–	–	–	–	–	–	–	–	–
Adjusted R^2^	0.8536	–	–	–	–	–	–	–	–	–
Predicted R^2^	0.7733	–	–	–	–	–	–	–	–	–
Adeq precision	10.6283	–	–	–	–	–	–	–	–	–
Quadratic		–	–	–	–	–	2.93E+07	306.48	336.43	350.92

#### ANOVA analysis

Using ANOVA, the effects of three independent parameters (pH, catalyst quantity, and lamp power) on the efficiency of hydrogen generation were ascertained. Using this method, the Fischer (*F*-test) test was applied at a 95% confidence level to determine the statistical significance of the quadratic effects of each component on the replies. The significance of model terms grows when fisher values (*F*) in the model rise, but *p*-values should fall concurrently. Meaningless words are defined as model variables with a significance level larger than 0.05 and are eliminated from the model. The quality of the fitted models was assessed using the coefficient of determination (*R*^2^). To attain an acceptable level of model fit, it must be around 1.0^36,49^. Additionally, the regression coefficient (*R*^2^), adjusted regression coefficient (Adj. *R*^2^), projected multiple determination coefficient (Pre. *R*^2^) and predicted residual error sum of squares (PRESS) were used to assess the model's appropriateness. Certain statistical values must be ascertained to test the model's suitability, including the correlation coefficient (*R*^2^), adjusted coefficient of determination (Adj. *R*^2^), predicted coefficient of determination (Pre. *R*^2^), adequate precision (Adeq Precision), coefficient of variation (CV), and predicted residual error sum of squares (PRESS). The statistical values needed to determine if the data in the adjusted model were appropriate were provided in Table 5 for hydrogen production efficiency.

The model had a very high regression coefficient *R*^2^ value of 0.9230. This demonstrated that the response variables in any variable in the experimental design could be determined using the quadratic regression model that was employed in the model. The near proximity of *R*^2^ and Adj. *R*^2^ values demonstrated the compatibility of the proposed model with the experimental data^50^. An adjuvant *R*^2^ value of 0.8536 was discovered. The *R*^2^ and Adj. *R*^2^ values differed by 0.06, indicating that the model prediction values were in excellent agreement with the actual hydrogen production yields found in the experimental investigations. The computed value of 0.08 was obtained by comparing the adjusted coefficient of determination (Adj. *R*^2^) with the projected coefficient of determination (Pre. *R*^2^). This difference was less than 0.2. This demonstrated the suitability of the model developed for the hydrogen generation efficiency computation. The ratio of signal to noise is measured by adequate sensitivity (Adeq Precision). The accuracy of the model should be valid if this number is higher than 4. The value of 10.6283 was discovered to be the Adeq Precision value of adequate sensitivity for hydrogen generation efficiency, which provides the needed value for model fit.

#### Parameter effects on hydrogen generation

**Figure 14:**
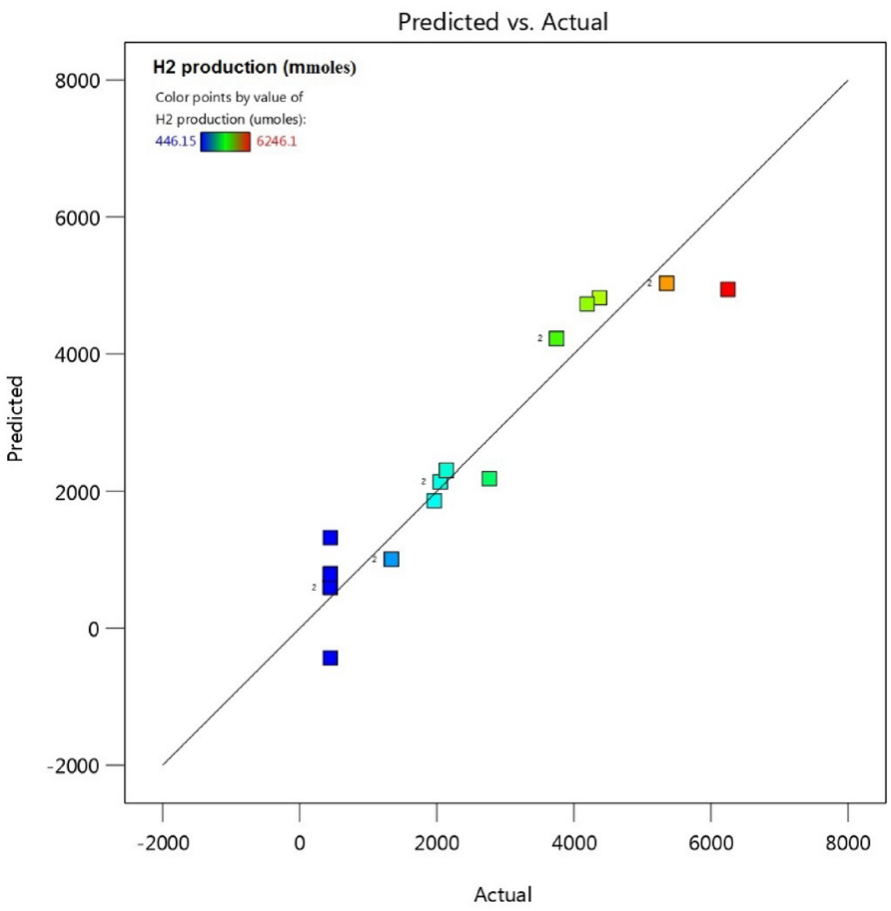
Comparison between the expected and actual outcomes.

**Figure 15:**
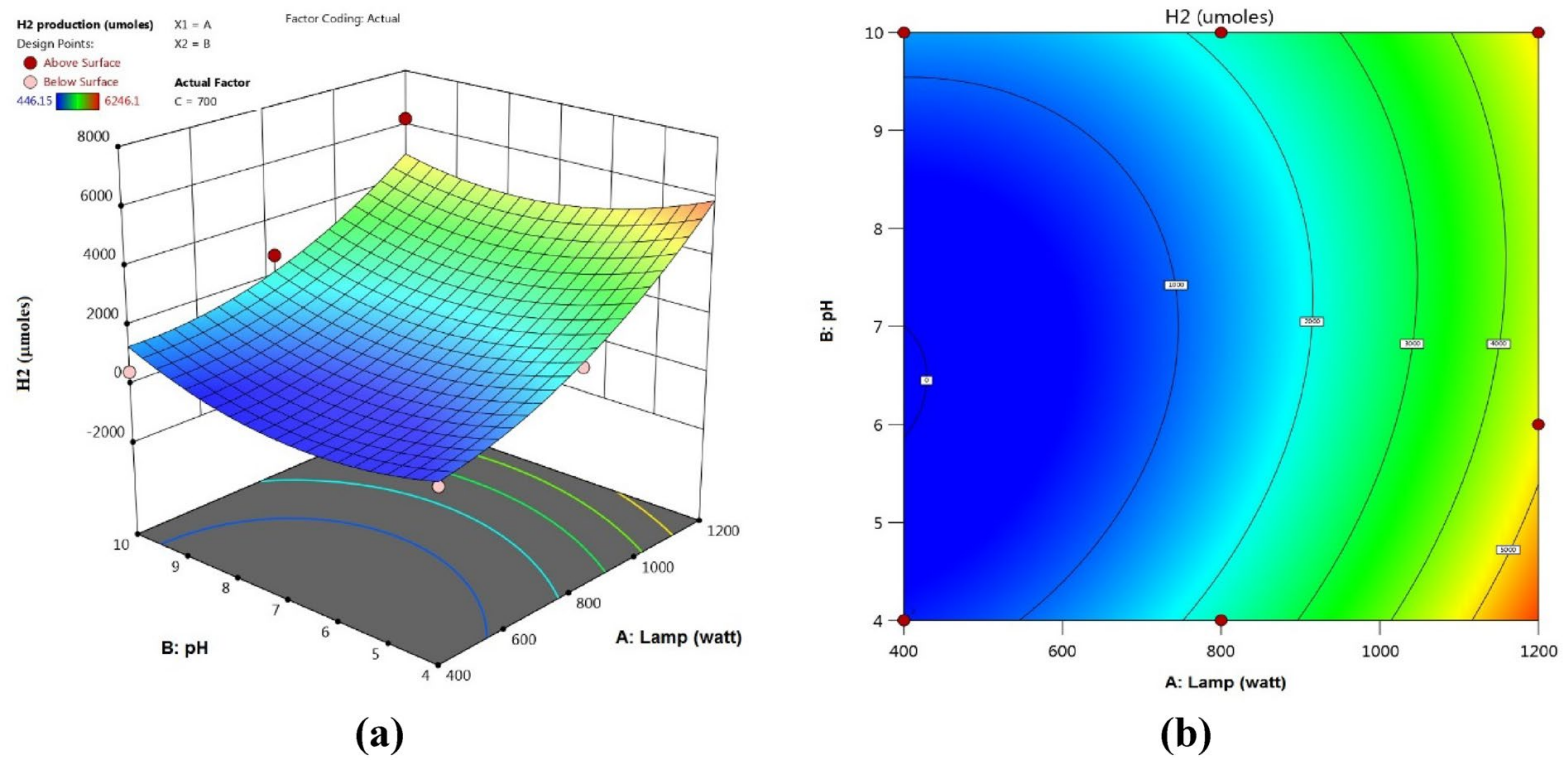
(**a**) 3D response surface plots, (**b**) contour plots: 2D influences of lamp power (W) and pH.

**Figure 16:**
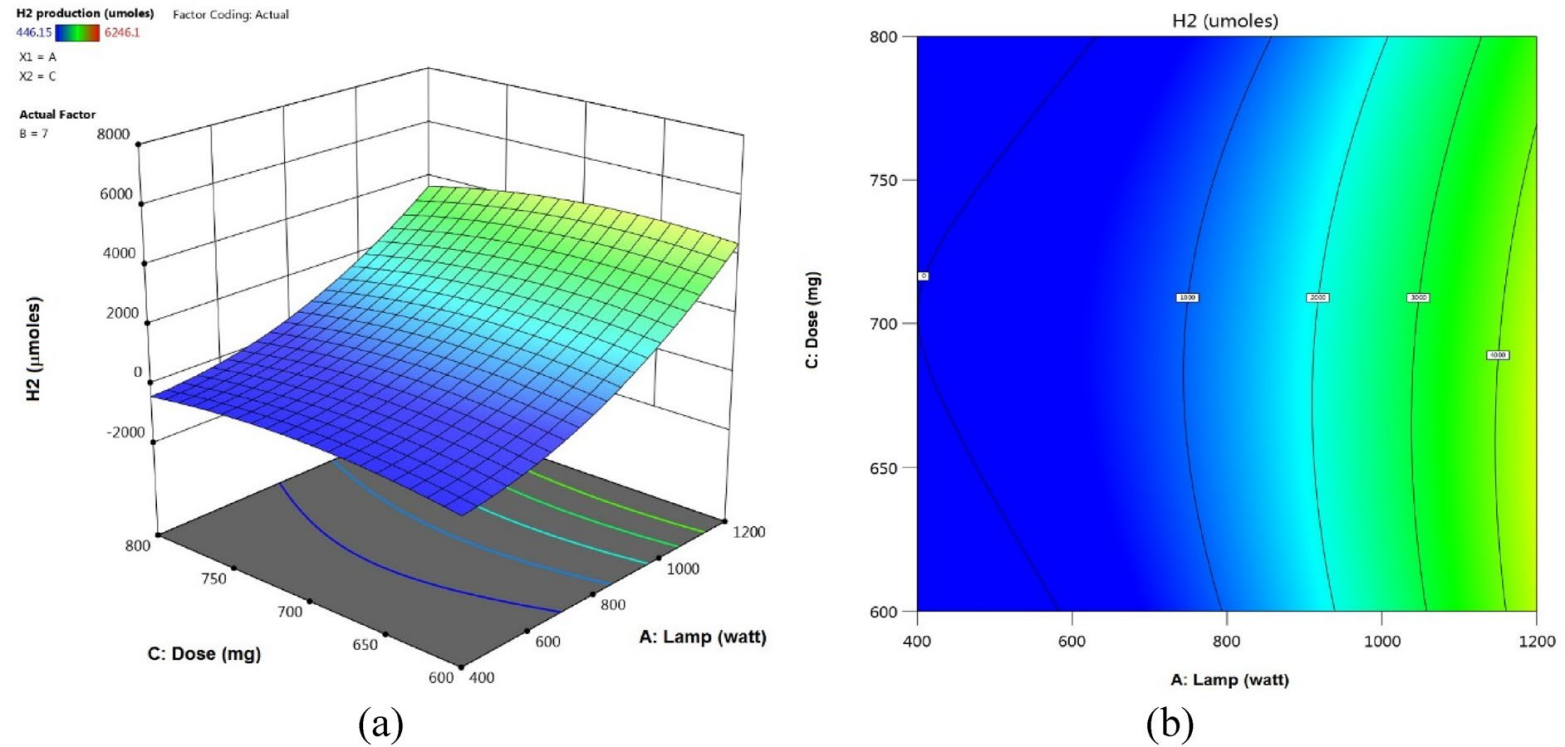
(**a**) 3D response surface plots, (**b**) contour plots: 2D influences of lamp power (W) and dose (mg).

**Figure 17:**
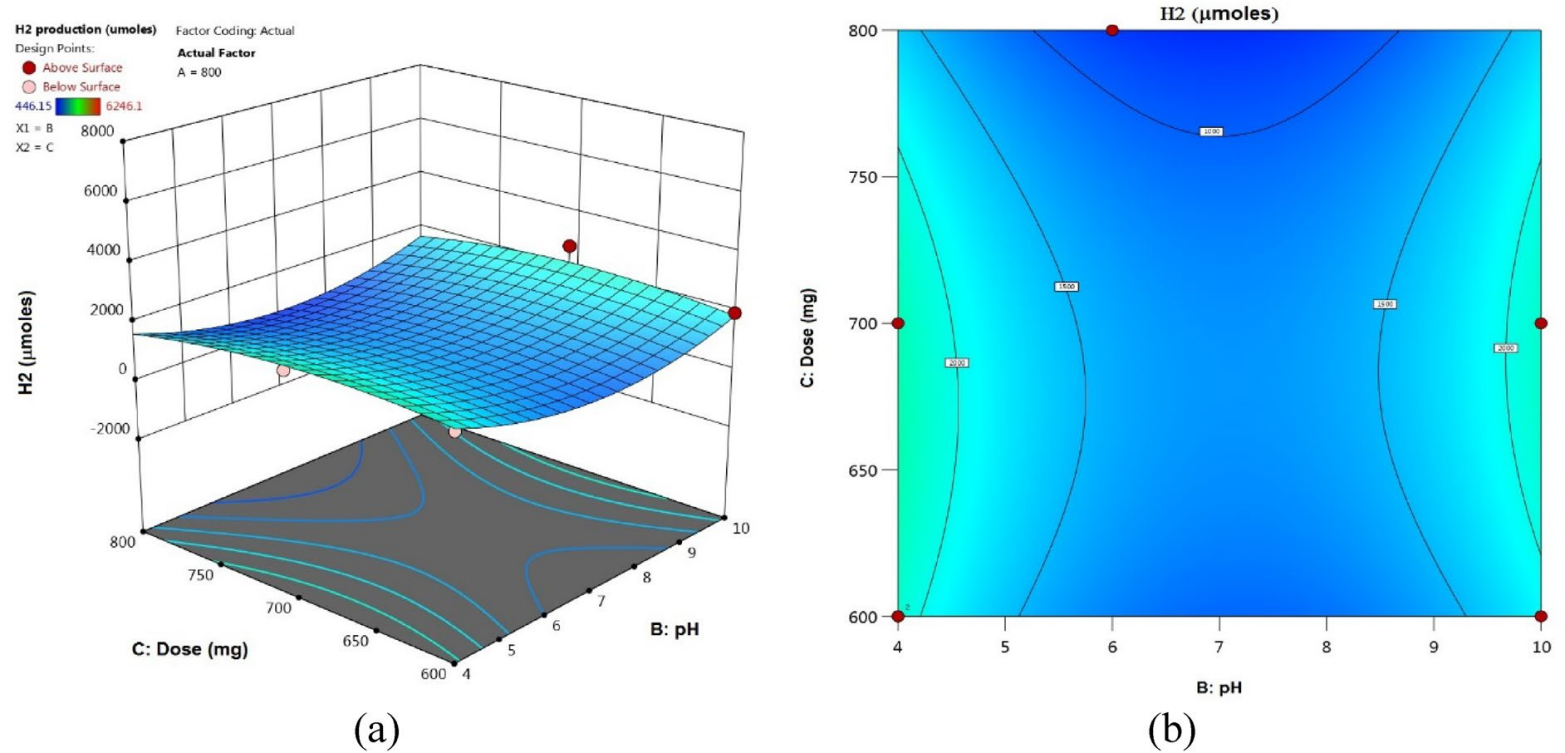
(**a**) 3D response surface plots, (**b**) contour plots: 2D Influences of pH and dose (mg).

Dependent variables are used to determine how independent factors affect the system (outputs). As a result, identifying the dependent variables is crucial. Three processes make up response variable modelling (RSM): (1) designing the experiment, (2) gathering data, and (3) building response variable prediction models based on research characteristics^51^. Applying the actual values of the model equation for H_2_ generation, predicted outcomes were derived. Figures 14, 15, 16, 17 showed the correlation between these outcomes and the test results that were actually obtained. Figure 14 shows that there was a fair degree of agreement between the observed H_2_-generated volume and the predicted H_2_-generated volume by the model.

Figures 15, 16, 17 included the 3D and 2D response surface graphs plotted against the electrolysis time, electrode voltage, and catalytic quantity of H_2_ generation. As seen in Fig. 15, the power light had a greater influence on the quantity of H_2_ produced than the pH did. An increase in lamp power from 400 to 1200 W led to an increase in hydrogen generation while the catalyst quantity remained the same. While the amount of catalyst remained constant, it was seen that the amount of hydrogen rose when the pH was raised from 4 to 10. The amount of hydrogen produced remained relatively constant when the amount of catalyst was increased from 600 to 800, as Fig. 16 illustrates. Figure 17 illustrates how an increase in pH might result in a greater generation of hydrogen. At pH 10, the volume of hydrogen grew steadily and reached 6246.096 µmol.

### Optimization and validation

The Design Expert 13 package program was utilized to determine the optimal parameter values for achieving maximum hydrogen generation, after an analysis of the effects of the parameters on hydrogen production. With the use of the model equations derived from the experimental data, optimization seeks to determine the optimal values of the independent variables by the intended response conditions.

**Figure 18:**
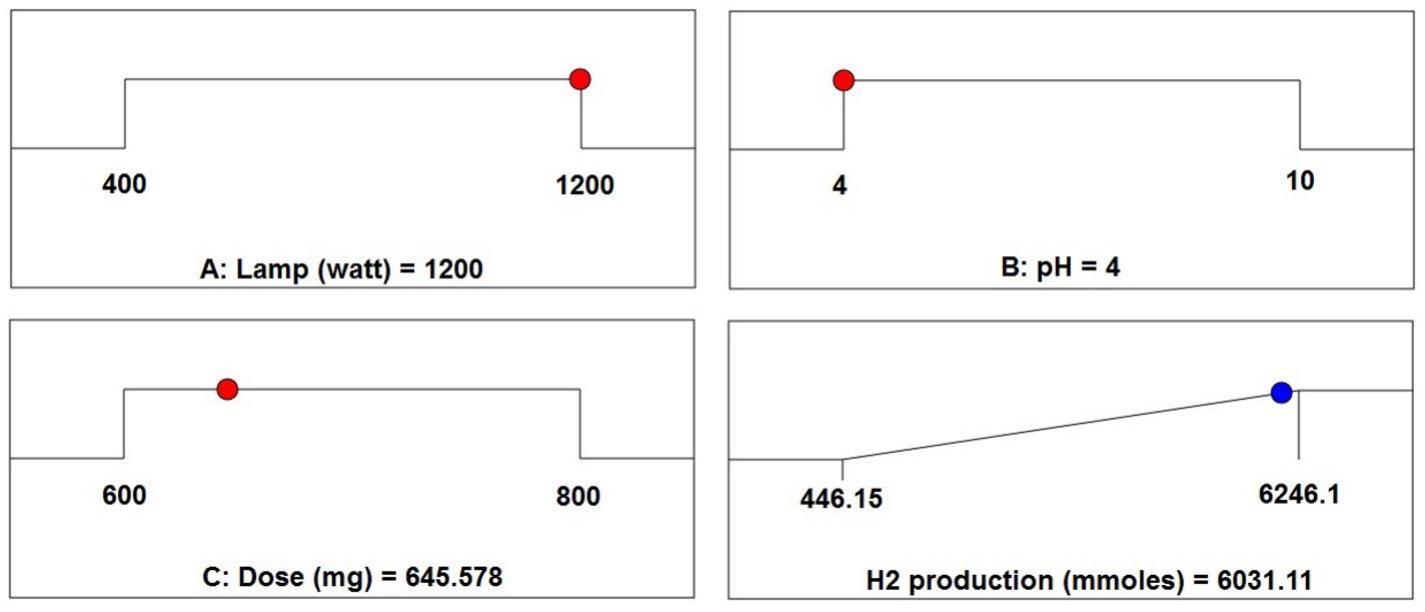
Optimization results of RSM showed the desirability equal 0.963 for the solution 1 out of 80.

The independent variables of lamp power (W), pH, and catalyst quantity (mg), which were judged to be useful parameters in hydrogen generation by the photocatalytic water splitting technique, were optimized to ascertain the amount of hydrogen generated. Figure 18 displays the optimal parameters of the RSM model. Validation experiments were conducted for the optimal values obtained as a consequence of the analysis to assess the dependability of the results obtained. The validation experiments aim to make a comparison between the actual values acquired from the validation experiment and the optimization solution ideas. Table 6 listed the optimal parameter values that were attained together with the quantity of hydrogen that matched these optimum values. These results indicate that the D-Optimal design and the experimental investigation produced outcomes that were very consistent with one another since they were quite near to one another.

**Table 6:** Results of the projected and experimental hydrogen volumes are compared.

Lamp power	pH	Catalyst dosage	Value	Hydrogen volume (µmol)
1200	4.0001	645.578	Predicted	6031.11
			Experimental	6246.1
			Error %	3.44

## Conclusions

In the present study, a visible light-mediated Ag-La-CaTiO_3_ photocatalyst was synthesized and its suitability for enhancing water splitting was investigated within a photocatalytic reactor. Different characterization techniques such as DRS, FTIR, XRD, XPS, EDX, SEM, TGA, DRS and BET was used to study the morphological and optical properties of the synthesized The Ag-La-CaTiO_3_ photocatalyst. The photocatalytic activity of the resulting Ag-La-CaTiO_3_ photocatalyst was examined under simulator visible light source, metal halide lamp with power of 1200-W. According to the Tauc plot, the optical characteristics of the Ag-La-CaTiO_3_ photocatalyst showed that the band gap was less than that of CaTiO_3_ (i.e., 3.002 eV for Ag-La-CaTiO_3_ and 3.6 eV of CaTiO_3_). Several parameters including different photocatalyst dosages, Different pH and different light intensity was used to achieve the optimal conditions of H_2_ production. The highest H_2_ yield (6246.09 μmol) was attained by using 700 mg of Ag-La-CaTiO_3_ photocatalyst, pH 10 and under 1200 W of irradiation. Finally, the RSM D-Optimal model was carried out to design, optimized the experiment and suggested that; the optimal conditions were pH 4, Ag-La-CaTiO_3_ dose of 645.578 mg and light intensities of 1200 W, which yield 6031.11 μmol of H_2_.

### Supplementary Information

Supplementary Figure S1.

## Data Availability

The corresponding author of the study can provide access to the datasets utilized in this inquiry upon request.
