# Supplementary material for: Experimental, predictive and RSM studies of H2 production using Ag-La-CaTiO3 for water-splitting under visible light
Source: Sci Rep. 2024 Jan 10;14:1019. doi: 10.1038/s41598-024-51219-z (PMC10781765; doi:10.1038/s41598-024-51219-z)
Supplement: Supplementary file 1 — Supplementary Figure S1. [file 41598_2024_51219_MOESM1_ESM.docx]

**Experimental, predictive and RSM studies of H_2_ production using Ag-La-CaTiO_3_ for water-splitting under visible light**

Safaa Ragab, Marwa R. Elkatory, Mohamed A. Hassaan, Ahmed El Nemr^*^

1. 3.033 mL of TTIP was added to 20 mL of ethanol with vigorous stirring for 30 min.

2. 5 mL of citric acid was added, stirring for 30 min.

(Solution A)

3. Mixed stoichiometrically solution (3% mole) of Ca(NO_3_)_2_, La(NO_3_)_2_ and Ag(NO_3_)_2_ (Solution B) was added to Solution A with keeping stirring at 50 °C.

4. The formed yellow solution was drying for 12 h at 50 °C. Excess organic chemicals and nitric acid were eliminated by burning it using a self-spread method.

5. The burning remains were calcined at 850 °C for 10 h to obtain Ag-La-CaTiO_3_.

H_2_ production

Light source


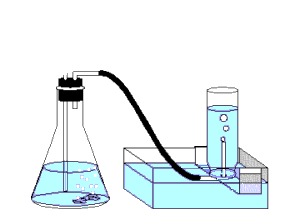


O_2_ Trap

Ag-La-CaTiO_3_

Fig. S1. Schematic of synthesis procedures of Ag-La-CaTiO_3_ photocatalyst for H_2_ production.
